# Treatment outcomes of patients with MDR-TB in Nepal on a current programmatic standardised regimen: retrospective single-centre study

**DOI:** 10.1136/bmjresp-2020-000606

**Published:** 2020-08-12

**Authors:** Samiksha Ghimire, Samriddhi Karki, Bhagwan Maharjan, Jos G W Kosterink, Daan J Touw, Tjip S van der Werf, Bhabana Shrestha, Jan-Willem Alffenaar

**Affiliations:** 1 Clinical Pharmacy and Pharmacology, University of Groningen Faculty of Medical Sciences, Groningen, The Netherlands; 2 Tuberculosis Unit, Nepal Anti-Tuberculosis Association/German Nepal TB Project, Kathmandu, Nepal; 3 Groningen Research Institute of Pharmacy, Department of Pharmaceutical Analysis, University of Groningen, Groningen, Groningen, the Netherlands; 4 Infectious Diseases Service and Tuberculosis Unit, University of Groningen Faculty of Medical Sciences, Groningen, The Netherlands; 5 Faculty of Medicine and Health, School of Pharmacy and Westmead hospital, University of Sydney, Sydney, New South Wales, Australia

**Keywords:** tuberculosis, respiratory infection

## Abstract

**Objectives:**

The objectives of this study were to evaluate treatment in patients on current programmatic multidrug-resistant tuberculosis (MDR-TB) regimen and verify eligibility for the 9-month regimen and therapeutic drug monitoring (TDM).

**Methods:**

We performed a retrospective chart review of patients with MDR-TB receiving standardised regimen at the German Nepal TB Project Clinic, Nepal, between 2014 and 2016. Eligibility for the 9-month regimen and indications for TDM were evaluated.

**Results:**

Out of 107 available patients’ medical records, 98 were included. In this centre, the MDR-TB treatment success rates were 69.0% in 2015, 86.6% in 2016 and 86.5% in 2017. The median time to sputum smear conversion was 60 days (60–90 *IQR*) and culture conversion was 60 days (60–90 *IQR*). Observed side effects did not impact treatment outcomes. No difference in treatment success rates was observed between patients with predisposing risk factors and those without. Only 49% (36/74) of patients were eligible for the 9-month regimen and 23 patients for TDM according to American Thoracic Society guideline criteria.

**Conclusions:**

Nepalese patients with MDR-TB on ambulatory care had good treatment outcome after programmatic treatment. Implementation of the new WHO oral MDR-TB treatment regimen may further improve treatment results. The 9-month regimen and TDM should be considered as part of programmatic care.

**Key messages**

**What is the key question?**

- The study evaluated treatment in patients on current programmatic multidrug-resistant tuberculosis (MDR-TB) regimen and verified eligibility for the 9-month regimen and therapeutic drug monitoring (TDM).

**What is bottom line?**

- The standardised MDR-TB regimen appears well tolerated in patients; none of the adverse events resulted in a change of regimen. Implementation of the new WHO oral MDR-TB treatment regimen may further improve treatment results. The 9-month regimen and TDM should be considered as part of programmatic care.

**Why read on?**

- The study provides complete treatment outcome data of patients with MDR-TB along with all significant clinical variables and drug susceptibility testing results (phenotypic and genotypic) make it relevant and provide a data-rich description of patients with MDR-TB treated in an outpatient setting.

## Introduction

Tuberculosis (TB) is the leading infectious disease caused by *Mycobacterium tuberculosis* that kills more people than HIV/AIDS every year. In 2018, an estimated 10 million people developed TB and 1.2 million died from it.^1^ Multidrug-resistant TB (MDR-TB), a form where the infecting strain is resistant to two important first-line drugs (isoniazid and rifampicin), has created havoc in TB control and has greatly hampered the TB elimination process.

Later generation fluoroquinolones (levofloxacin and moxifloxacin) are key drugs in both standardised (20–24 months duration), short course 9-month and all-oral 20-month regimens in use for treating MDR-TB.^1 2^ Levofloxacin and moxifloxacin containing regimens had similar treatment outcomes in four published clinical trials.^2–6^ Although the role of gatifloxacin was believed to be critical in the success of the short 9-month regimen studied in Bangladesh, Niger and Cameroon, 7–9 the drug is not included in the new WHO consolidated guidelines for the treatment of drug resistance TB.^1^ With the new all-oral 20-month regimen which includes drugs like bedaquiline and delamanid, the overall proportion of adverse events was reasonably low compared with standardised 20–24 month regimen with injectable agents.^10 11^ More evidence is needed to assess the tolerability of the new all-oral regimen, especially in settings with limited resources where short 9-month regimen has demonstrated to be safe and effective.^12^ Furthermore, linezolid, a narrow therapeutic index drug, will be frequently used in the all-oral regimen; therefore, balancing efficacy and toxicity is crucial.^13 14^ Therapeutic drug monitoring (TDM) can be used as a tool to ensure target attainment while preventing toxicity.^1 10 13 15–17^

In the light of available evidence, it’s important to explore the link between various risk factors like diabetes mellitus, HIV, advanced age, low body weight and adverse events and treatment outcomes in patients under a standardised regimen for MDR-TB treatment in a local setting.^1 2^ Therefore, the aim of this study was to evaluate treatment outcomes of patients with MDR-TB on current programmatic regimen. Furthermore, this study aims to explore the eligibility of patients with baseline resistance to more than one first-line drug for the shorter 9-month regimen and evaluate if TDM could be recommended.

## Methods

### Study population and study design

A retrospective chart review was performed for all patients with MDR-TB receiving levofloxacin as part of their standard MDR-TB regimen enrolled for treatment at the Nepal Anti-Tuberculosis Association/German Nepal TB Project (NATA/GENETUP), Kathmandu, Nepal, between April 2014 and December 2016. Patients received 750–1000 mg levofloxacin one time a day. The other drugs included in the regimen were kanamycin at a median dose of 750–1000 mg, cycloserine and ethionamide both at one time a day dose of 500–750 mg and pyrazinamide at 1200–1600 mg one time a day dosing. Patients with missing records/incomplete information were excluded, and those who had pre-XDR TB (Extensively drug resistant) (with either fluoroquinolone resistance or resistance to second-line injectable agents) were also excluded. In the GENETUP clinic, all patients were tested for HIV and diabetes mellitus as a standard procedure. All patients with HIV were on antiretroviral therapy (tenofovir, lamivudine and efavirenz/dolutegravir-based regimen). Similarly, patients with diabetes mellitus were on hypoglycaemic agents.

#### Model of care and pharmacovigilance

Primarily, patients were treated as outpatients from the start of treatment. Residential facilities (included meals and monthly allowance of US$1.5) were provided to impoverished patients, those at the risk of treatment non-adherence and treatment failure. Due to limited room capacity, patients were qualified eligible for residential facilities on a case-by-case basis giving priority to those in dire need. This allowed healthcare providers at NATA/GENETUP to closely monitor and manage MDR-TB treatment in vulnerable subpopulations. For patients in ambulatory care, adherence was ensured by keeping contact information of two additional patient relatives along with their home address details. In some cases, community members were contacted if both patient and patient parties were unreachable. Since TB is considered a public health threat in Nepal, seldom, police authorities were involved to provide counselling to non-adherent patients.

Medication safety was evaluated by recording adverse reactions in a standardised adverse effect monitoring form, every month until treatment completion. On daily visits to GENETUP clinic for DOTS (Directtly Observed Treatment, Short Course) facilities, patients were given the opportunity to report serious side effects. Then, based on complaints and serum biochemistry results, offending agents were either discontinued for a period of time or replaced with other available drugs in the regimen.

#### Patient and public involvement

This being a retrospective study, patients were not involved.

### Culture, DST, clinical variables and outcome

Before initiation of treatment, two sputum samples were collected from patients for culture and second-line drug susceptibility testing (DST), and subsequently every month until 8 months followed by 10, 12, 16, 18, 20, 22 and 24 months for treatment monitoring by smear microscopy and culture on Löwenstein-Jensen media. Phenotypic first-line and second-line DST was carried out at the WHO critical concentrations.^18^ The genotypic DST (GenoType MTBDR*plus*) molecular line probe assay was used for diagnosis of isoniazid (*kat*G and *inh*A gene), and rifampicin (*rpo*B gene) resistance. For second-line drugs, molecular line probe assay (Hain Life Science, *Geno*Type MTBDR*sl*) was used to identify resistance pattern to fluroroquinolones (*gyr*A gene), and aminoglycosides (*rrs* gene) and ethambutol (*emb*B gene). Furthermore, the study evaluated impact of initial ethambutol resistance on MDR-TB treatment outcomes. Clinical variables included age, body weight at admission, body weight after 8 months of treatment, body weight below 35 kg, gender, HIV status, comorbidities, prior anti-TB therapy and presence of cavitary lesions. To evaluate the safety of the multidrug regimen, all recorded adverse events and lab test results at three different time periods (baseline, third month and 5–8 months of treatment) were retrieved from the medical records.

The MDR-TB treatment outcomes are defined based on WHO guidelines adopted by the NTP (National Tuberculosis Programme), Nepal.^19^ This study used Laserson’s recommendations to note the treatment outcomes where deaths included all deaths irrespective of the cause during the course of MDR-TB treatment.^20^ Successful treatment outcome relates to cure and treatment completion whereas unsuccessful outcome is characterised by failure, death, relapse, loss to follow-up or transfer out.

DST results on first-line and second-line drugs were obtained to evaluate potential eligibility of patients in a shorter 9-month MDR-TB regimen. Furthermore, an official clinical practice guideline from the American Thoracic Society was used to evaluate patients eligible for TDM. Criteria for TDM were patients with gastrointestinal problems that increase risk of malabsorption, concurrent HIV infection, impaired renal clearance, diabetes or patients not responding to MDR-TB therapy by the third month.^16 21^

### Statistical analysis

The association of variables with treatment outcomes (successful/unsuccessful) was studied using univariate logistic regression analysis (SPSS, V.23.0 IBM Corp., New York, USA). In the univariate analysis, variables with a p value of <0.25 were selected for entry in the final multivariate model. A p value of <0.05 was considered significant. Categorical data were expressed in frequencies and percentages whereas continuous variables were presented as median and IQR. Depending on the distribution of continuous variables, non-parametric tests were used for calculation of p values, where applicable.

## Results

**Figure 1:**
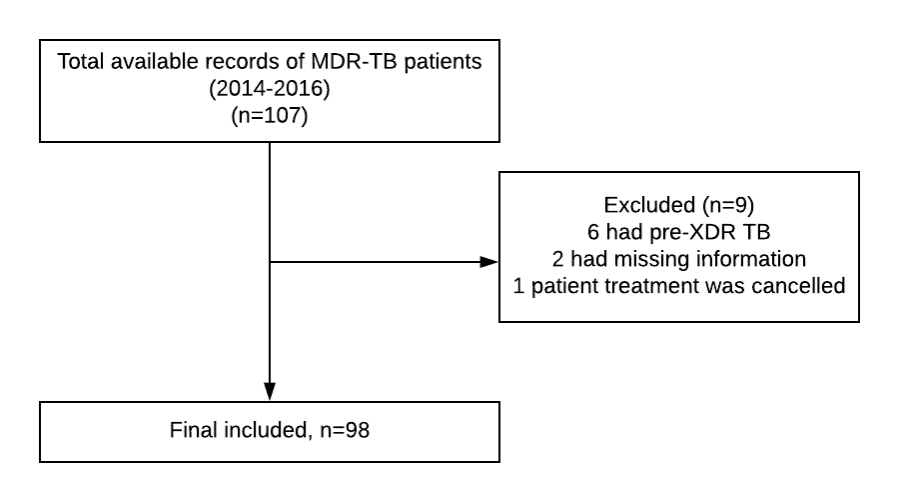
Flow chart of the study population. MDR-TB, multidrug-resistant tuberculosis.

Out of 107 available patients’ medical records, a total of 98 MDR patients were included in this retrospective chart review (see figure 1). Baseline characteristics of these 98 patients are summarised in table 1. Patients were treated for a median period of 20 months (20–24; min, max). Pulmonary TB was the most common diagnosis in 93 (94.8%) patients; 5 (5.1%) had extrapulmonary TB. The majority of the patients—87 (88.7%)—had prior anti-TB therapy. Among these retreated patients, 34 (39.1%) had failed on a 6-month treatment regimen with first-line drugs and 19 (21.8%) had failed on the 8-month retreatment regimen with first-line drugs including streptomycin (table 2).

**Table 1:** Demographic and clinical characteristics of included patients (n=98)

Age, years	29 (22–40)
Body weight at admission, kg	48 (44–58)
Body weight after 8 months of treatment, kg (n=85)	56 (47–60)
Gender, male (n=98)	56 (57.1)
Comorbidity (n=80)	
HIV	6 (7.5)
Diabetes mellitus	6 (7.5)
Seizure disorder	4 (5.0)
Cardiovascular disease	1 (1.3)
Active hepatitis/cirrhosis	1 (1.3)
Osteoporosis	1 (1.3)
Gastric ulcer	1 (1.3)
Diagnosis (n=96)	
Sputum	92 (95.8)
Others (pleural fluid, lymph node aspirate)	4 (4.2)
Radiographic findings (n=83)	
Cavitary lesions	7 (8.4)
Bilateral pulmonary involvement with cavitary lesions	10 (12.1)
Bilateral pulmonary involvement without cavitary lesions	31 (37.4)
Non-cavitary non-bilateral pulmonary involvement	33 (39.8)
Normal chest finding	2 (2.4)
Drug resistance, phenotypic testing	
Streptomycin (n=14)	13 (92.9)
Isoniazid (n=24)	24 (100)
Rifampicin (n=88)	88 (100)
Ethambutol (n=74)	39 (52.7)
Drug resistance, genotypic testing	
Isoniazid (n=9), InhA wildtype and katG Mut 1	9 (100)
Rifampicin (n=9), rpoB (mutation-3)	9 (100)
Ethambutol (n=74), emB-Mut1	38 (51.4%)

Categorical data are expressed in frequencies and percentages whereas continuous variables are presented as median and IQR.

**Table 2:** Patients with prior tuberculosis (n=87)

Causes of failure	Cases	Percentage
Treatment after failure of category I	34	39.1
Treatment after failure of category II	19	21.8
Treatment after lost to follow-up	1	1.1
Relapse	33	37.9
Total	87	100

Category I included treatment with isoniazid, rifampicin, pyrazinamide and ethambutol whereas, category II included treatment with HRZE (Isoniazid, Rifampicin, Pyrazinamide, Ethambutol) plus streptomycin.

The median time to sputum smear conversion (n=75) was 60 days (60–90 *IQR*) and culture conversion (n=81) was also 60 days (60–90 *IQR*); two patients remained culture positive after intensive treatment characterised by the growth of NTM (Nontuberculous mycobacteria) later on (8th and 10th month), respectively. Treatment outcomes of the study population (n=98) were as follows: 73 (74.5%) patients were cured, 12 (12.2%) completed the treatment, 3 (3.1%) were lost to follow-up, 4 (4.1%) were transferred out, 5 (5.1%) died and 1 (1%) failed MDR-TB treatment. Among five deaths, three died during the first 2 months of TB treatment, one committed suicide at month 14 of treatment and one critically ill child died at an intensive care unit during the first month of TB treatment. In our study, 86.7% had successful outcome (n=98) based on standards proposed by Laserson *et al*.^13 20^ In a univariate analysis, only days to culture conversion was significantly (p=0.002) associated with treatment outcomes. Results of univariate analysis are available in online supplementary material.

10.1136/bmjresp-2020-000606.supp1 Supplementary data

The lab test results at three different time periods in patients with MDR-TB are summarised in table 3. The majority of patients had hyperuricaemia. Based on alanine amino transferase (ALT), aspartate transaminase and bilirubin levels, only one patient had to temporarily interrupt the treatment due to hepatotoxicity. The levels of ALT did not escalate more than 3–5 times the upper limit of normal in the majority of patients. Moreover, information on side effects of medical treatment was available for 97 patients. Seven (7.2%) out of 97 patients did not experience any side effects. Side effects observed in the remaining 90 (92.8%) patients are shown in table 4. Psychosis was the most common side effect leading to the withdrawal of cycloserine, which was then replaced by clofazimine. Hepatotoxicity led to the discontinuation of pyrazinamide in only one patient.

**Table 3:** Hepatic enzymes and renal function tests in patients with multidrug-resistant tuberculosis

	Normal reference level	At baseline	3 months	5–8 months	P value (baseline and 3 months)	P value (baseline and 5–8 months)
Hepatic enzymes						
Alanine amino transferase, IU/L	5.0–40.0	17.0 (12.0–23.5) (n=84)	12.0 (9.0–18.5) (n=77)	15.0 (10.0–22.3) (n=50)	0.004*	0.302*
Aspartate transaminase, IU/L	5.0–37.0	26.0 (18.0–38.0) (n=84)	27.0 (20.0–36.0) (n=77)	30.0 (21.0–42.0) (n=50)	0.516*	0.445*
Alkaline phosphatase, IU/L	65.0–305.0	180.0 (143.0–215.0) (n=83)	171.0 (136.0–212.0) (n=77)	179.5 (150.0–242.0) (n=47)	0.211*	0.488*
Bilirubin total, µmol/L	6.8–17.1	10.3 (8.6–11.9) (n=84)	10.3 (8.6–11.9) (n=77)	10.3 (8.6–11.9) (n=49)	0.694*	0.735*
Bilirubin conjugated, µmol/L	0.2–6.8	5.1 (3.4–5.1) (n=80)	5.1 (3.4–5.1) (n=74)	5.1 (3.4–5.1) (n=46)	0.868*	0.364*
Renal markers						
Creatinine, µmol/L						
Males n<124	53.0–123.8	70.7 (61.8–79.5) (n=86)	70.7 (61.8–79.5) (n=85)	79.6 (61.8–88.4) (n=82)	0.011†	0.005†
Females n<107	44.2–106.8					
Potassium, mmol/L	3.5–5	4.1 (3.8–4.4) (n=86)	4.0 (3.8–4.4) (n=84)	4.2 (3.9–4.5) (n=85)	0.760†	0.324†
Sodium, mmol/L	135–146	143.0 (140.0–144.50) (n=85)	144.0 (141.0–145.0) (n=84)	144.0 (141.0–145.0) (n=85)	0.006*	0.130*
Urea, mmol/L	3.6–16.0	6.24 (5.4–8.2) (n=83)	7.49 (5.7–10.4) (n=85)	6.78 (5.7–9.3) (n=82)	0.005*	0.125*
Uric acid, mmol/L M: n<0.42 F: n<0.35	0.20–0.41	0.41 (0.33–0.53) (n=78)	0.48 (0.41–0.59) (n=78)	0.51 (0.39–0.59) (n=80)	0.006†	0.011†

Data are presented as median (IQR) for all continuous variables. Renal function was defined based on creatinine level. Creatinine level=132–194 µmol/L was considered impaired and >203 µmol/L was considered severely impaired; hyponatraemia=serum sodium below 135 mmol/L, severe hyponatraemia was less than 115 mmol/L; hypokalaemia=potassium less than 3.5 mmol/L; hyperkalaemia=potassium levels higher than 6 mmol/L. Hyperuricaemia=levels above the upper limit of normal for both males and females.

*Paired t-test.

†Wilcoxon signed-rank test.

**Table 4:** Side effects associated with the multidrug-resistant tuberculosis treatment

Side effects (n=90)	Offending drugs	n (%)
Arthralgia	Levofloxacin, pyrazinamide	63 (70)
Hypothyroidism	Ethambutol	50 (55.6)
Gastrointestinal symptoms	Ethambutol, ethionamide	41 (45.6)
Dizziness/vertigo	Kanamycin, levofloxacin	20 (22.2)
Hearing loss	Kanamycin	6 (6.7)
Clinical signs of hepatotoxicity	Pyrazinamide, ethionamide	2 (2.2)
Alopecia		1 (1.1)
Central nervous system		
Sleep disturbances/minor mood	Cycloserine, levofloxacin	15 (16.7)
Depression	Cycloserine	5 (5.6)
Psychosis	Cycloserine	4 (4.4)
Suicidal thoughts	Cycloserine, levofloxacin	2 (2.2)

Furthermore, we evaluated the proportion of patients with available DST results on first-line and second-line drugs for their potential eligibility in a shorter 9-month MDR-TB regimen. The results from both phenotypic and genotypic testing revealed that 52% of the patients with MDR-TB (n=74) had resistance to ethambutol. Based on results of this study, if ethambutol resistance were to be considered, only 48% of patients with MDR-TB would be eligible for shorter 9-month regimen. However, in programmatic settings like Nepal resistance to ethambutol is not taken into account because only fluoroquinolones and second-line injectables are considered important core drugs in a the shorter 9-month regimen. There is a documented evidence that the regimen fails only when the isolate is resistant to core drugs but not companion drugs (ethambutol, pyrazinamide or clofazimine).^12^ Therefore, patients had to meet three criteria to be eligible for a shorter 9-month regimen in Nepal: confirmed susceptibility to injectables, confirmed susceptibility to fluoroquinolines and low-level isoniazid resistance: *Inh*A mutations for isoniazid (patients with *kat*G mutations were disqualified and started on a longer 18–20 month regimen). Among 38 patients with MDR-TB who had initial resistance to ethambutol, all of them achieved favourable treatment outcomes (Laserson recommendations). Of 38 patients, 32 (84.2%) were deemed cure and 6 (15.8%) completed the treatment. However, ethambutol was not part of the longer 18–20 month MDR-TB regimen.

In our study, 23 patients categorised as high-risk group to poor MDR-TB treatment would have been eligible for TDM (table 5). Among these 23 patients: 6 patients had HIV, 6 had diabetes mellitus, 1 had liver cirrhosis, 1 had gastric ulcer and 1 elderly patient had extremely low creatinine clearance which is of relevance to levofloxacin pharmacokinetics; and 8 patients did not attain culture conversion in 90 days.

**Table 5:** Therapeutic drug monitoring (TDM) indications**

TDM indication (n=80)	N (%)	Effects on pharmacokinetics of anti-TB drugs
Diabetes mellitus	6 (7.5)	Diabetes induced gastroparesis leading to either delayed absorption, malabsorption or altered clearance of anti-TB drugs
Concurrent HIV infection	6 (7.5)	Reduced exposure of anti-TB drugs
Gastric ulcer	1 (1.3)	Interference with absorption of anti-TB drugs
Liver cirrhosis	1 (1.3)	Altered drug metabolism, prolonged effect of parent drug, reduced effect of prodrugs, increase in toxic metabolites
Impaired renal clearance	1 (1.25)	Over exposure of renally cleared drugs, increased toxicity, require dose reduction
Slow treatment response (n=73)	8 (11)	Possibly suboptimal exposure of anti-TB drugs due to interindividual variabilities

*Indications based on references 16 21.

TB, tuberculosis.

## Discussion

The study evaluated treatment results of current programmatic regimen before implementation of all-oral 18–20 month regimen which includes new drugs like bedaquiline and delamanid, at the TB referral clinic of Nepal.

We found a high treatment success rate of 86.7% for our MDR-TB cohort. In a previous study by Malla *et al*, 70% of the outpatients with MDR-TB were reported to be cured on a regimen containing ofloxacin.^22^ The improved outcome may be explained by the use of the levofloxacin instead of ofloxacin and early prescreening of patients for resistance to second-line drugs.^22^ However, results from our study should be interpreted with caution. First, patients who were classified as pre-XDR were excluded because they were put on a different regimen containing moxifloxacin and not levofloxacin. However, moxifloxacin should not be used if documented resistance to FQs (Fluoroquinolones). Second, our study is a single-centre study. Only patients managed in the GENETUP referral clinic were studied. This centre in the capital of Nepal is well equipped with molecular and phenotypic DST. In this centre, the treatment success rates for MDR-TB cases were 69.0% in 2015, 86.6% in 2016 and 86.5% in 2017. Owing to its high treatment success, the model of care implemented by the GENETUP clinic should also be adopted by other MDR-TB treatment sites in Nepal.

One of the striking observations from this study was that the majority of patients with MDR-TB had prior anti-TB therapy and 60% had failed a 6–8 month treatment regimen with first-line drugs and streptomycin. This is in contrast with the high success rate of drug-susceptible TB and not on par with the incidence of MDR-TB (15.4% of retreatment cases are MDR-TB based on drug resistance survey carried out in 2011/2012).^19^ Despite use of DOTS, these patients might have been patients with MDR-TB misdiagnosed as drug-susceptible TB due to unavailability of GeneXpert MTB/RIF and therefore put on an ineffective first-line regimen; or they might have acquired resistance to rifampicin/isoniazid during the course of treatment with first-line drugs; or they may have been reinfected with drug-resistant bacilli from a source patient having MDR-TB. In Nepal, the primary drugs resistance is high; treating patients who failed on category I treatment with addition of streptomycin might have resulted in higher failure of category II treatment. The routine drug resistance survey in Nepal showed a higher proportion of resistance to second-line drugs, with resistance to fluoroquinolones alone at 39.3% among patients with MDR-TB.^19^ This implies that 40% of the patients with MDR-TB in Nepal might require pre-XDR TB treatment. In 2017 alone, around 35.4% of confirmed MDR-TB cases were pre-XDR (91 pre-XDR cases among 257 diagnosed MDR-TB cases).^19^ In 2017, overall treatment success rate of pre-XDR TB was 58% and XDR TB was 61%. The death rate was quite high among XDR TB cases (39%) and encouragingly, there was no lost to follow-up among XDR TB cases. Since clinical diagnosis of pre-XDR TB is not possible, this calls for a massive scale up of genotypic susceptibility testing in decentralised TB treatment centres and subcentres, to adequately diagnose patients and start appropriate treatment.^23^ Moreover, as per local guidelines, previously diagnosed patients with TB and a typical TB chest X-ray failing on 6-month treatment regimen with first-line drugs and on the 8-month retreatment regimen with first-line drugs including streptomycin are put on MDR-TB treatment. This is because overall treatment outcomes of patients who fail both 6 and 8 month regimens are unacceptably poor if not placed on MDR-TB regimen. This might improve significantly in future as GeneXpert is being rolled out massively in Nepal, with a total of 55 GeneXpert centres at present.

The side effects of MDR-TB drugs in patients remain a major concern but most were managed without change of the MDR-TB regimen. Regarding eligibility of patients for the shorter 9-month MDR-TB regimen, it is unclear if patients with resistant strains to ethambutol would qualify. Van Deun *et al* argued that full susceptibility to ethambutol and isoniazid was not of paramount importance for patients to qualify for treatment with the shorter regimen.^24^ This could be problematic and result in inclusion of patients with different drug resistance profiles compared with those at trial sites in Niger, Cameroon and Bangladesh on which the 9-month regimen is based.^25^ In a recently published study by Piubello *et al*, successful outcomes were not affected by initial resistance to companion drugs (ethambutol, pyrazinamide and clofazimine).^12^ In line with their study, initial resistance to ethambutol did not seem to negatively affect treatment outcomes in our study.

Since January 2018, the NTP of Nepal has endorsed the 9-month shorter regimen for the treatment of MDR-TB with weight band moxifloxacin dosing (600 mg or 800 mg) in all TB treatment centres, with the support of the Damien Foundation. At present, resistance to ethambutol and isoniazid (due to shortage of test kits) is not considered while determining eligibility. Patients are eligible if deemed susceptible to fluoroquinolones and aminoglycosides. Between July 2018 and July 2019, 72% (31/43) of the registered MDR-TB patients were eligible for a shorter 9-month treatment regimen in the GENETUP clinic. The remaining 28% received programmatic standardised MDR-TB regimen with injectable agents (kanamycin). Encouragingly, with the implementation of all-oral 18–20 month regimen, the trend is shifting with more patients being switched to all-oral regimen over shorter 9-month regimen containing injectable agents. The treatment outcome results from the shorter 9-month regimen in a high-incidence, high-fluoroquinolones-resistant setting like Nepal will be communicated to the TB community likely in 2020.

In relation to drug exposure, 30% of the patients at the GENETUP clinic who were enrolled for a prospective pharmacokinetic study between 2016 and 2017 did not meet the established levofloxacin target for efficacy on one time a day 750–1000 mg dosing.^26^ These Nepalese patients would have benefitted from dose adjustments based on TDM.^26–29^ In the official clinical practice guidelines,^21^ TDM is recommended to all patients on second-line drugs, but in settings with high TB endemicity these recommendations have yet to see the daylight.^30^ MDR-TB treatment centres in countries such as the Netherlands, Sweden, Germany and USA routinely employ plasma/serum TDM to estimate C_max_ and AUC_0-24_ (Area under the concentration-time curve) of core drugs like moxifloxacin, linezolid and aminoglycosides, among others which has helped ensure adequate drug exposure and minimise associated toxicity. The treatment outcomes for MDR-TB in these centres are comparable to those reported globally for drug-susceptible TB (above 75%).^30^ Therefore, it is about time that TDM is incorporated as a part of programmatic care also in settings like Nepal.

This study has several limitations. First, being a retrospective study from a single centre, the generalisability is limited. However, due to the excellent programmatic care in this centre, it could serve as role model for other centres in the country and region. Second, lack of difference in treatment outcomes between predisposing risk factors and non-risk factors groups could be due to the low statistical power and high proportion of cure and completion rates compared with lost to follow-up, death and relapse, due to unavailability of patient information in the latter group. Pooling individual patient data from several treatment outcome studies as has been done by the Canadian team led by Menzies would likely improve the statistical power for future studies to detect a difference in response.^31^ It is also imperative to have a standardised data collection strategy for wider generalisation of results. Nevertheless, our study has a major strength. The availability of complete treatment outcome data along with all significant clinical variables and DST results (phenotypic and genotypic) makes it relevant and provides a data-rich description of patients with MDR-TB treated in an outpatient setting. The study will likely be used to compare with results from the 9-month and all-oral MDR-TB regimens.

In conclusion, the standardised MDR-TB regimen appears well tolerated in patients; none of the adverse events resulted in a change of regimen. Implementation of the new WHO oral MDR-TB treatment regimen may further improve treatment results. For patients resistant to fluoroquinolones or second-line injectables, all-oral regimen might be the best option and for those without resistance to fluoroquinolones or second-line injectables, the shorter regimen is indicated. The 9-month regimen and TDM should be considered as part of programmatic care.
