# Supplementary material for: Treatment outcomes of patients with MDR-TB in Nepal on a current programmatic standardised regimen: retrospective single-centre study
Source: BMJ Open Respir Res. 2020 Aug 12;7(1):e000606. doi: 10.1136/bmjresp-2020-000606 (PMC7430340; doi:10.1136/bmjresp-2020-000606)
Supplement: Supplementary data [file bmjresp-2020-000606supp001.pdf]

Supplementary material:

Factors associated with treatment outcome at the end of treatment using univariate logistic regression analysis

Independent variables (n=98)		Treatment outcome		OR (0.95% CI)	p-value
		Successful (n=85)	Unsuccessful (n=13)		
***Age**		29 (22-40)	33.5 (25- 44.7)	0.97 (0.95-1.04)	0.896
***Body weight at admission**		48 (45-57.5)	47.0 (36.2-60.5)	1.03 (0.97-1.09)	0.347
***Body weight after 8 months (n=85)**		56 (47-60)	43.2 (10.5-NA)	1.08 (0.98-1.18)	0.140
***Levofloxacin dose, mg/kg**		15.6 (13.5-17.0)	16 (12.92-18.35)	0.99 (0.80-1.22)	0.929
**Gender**					
	Male	48 (56.5)	7 (58.3)	0.93 (0.27-3.15)	
	Female	37 (43.5)	5 (41.7)	Ref	0.903
**Prior anti-TB therapy**					
	No	8 (9.4)	2 (16.7)	Ref	0.446
	Yes	77 (90.6)	10 (83.3)	1.92 (0.35-10.36)	
***Days of sputum conversion (n=75)**		90 (60-105)	45 (30-112.5)	1.01 (0.98-1.04)	0.321
***Days of culture conversion (n=81)**		90 (60-90)	30 (30-37.5)	1.09 (1.03-1.15)	0.002
**Alcohol abuse (n=84)**					
	No	62 (82.7)	8 (88.9)	Ref	
	Yes	13 (17.3%)	1 (11.1%)	1.67 (0.19-14.51)	0.639
**Chest Radiography (n=80)**					
	Cavitary lesion	5 (5.9)	1 (8.3)	0.80 (0.07-8.75)	0.855
	Bilateral pulmonary involvement with cavitary lesion	10 (11.8)	1 (8.3)	1.60 (0.16-16.13)	0.690
	Bilateral pulmonary involvement	32 (37.6)	2 (16.7)	2.6 (0.43-15.12)	0.300
	Non cavitary non-bilateral pulmonary involvement	25 (29.4)	4 (33.3)	Ref	

Data are presented as n (%), unless otherwise stated: *median (interquartile ranges). OR: odds ratio; CI: confidence intervals
